# Exploratory analyses of biomarkers in blood and stratum corneum in patients with atopic dermatitis

**DOI:** 10.1097/MD.0000000000031267

**Published:** 2022-10-21

**Authors:** Ayano Maruyama, Risa Tamagawa-Mineoka, Hiromi Nishigaki, Koji Masuda, Norito Katoh

**Affiliations:** a Department of Dermatology, Graduate School of Medical Science, Kyoto Prefectural University of Medicine, Kyoto, Japan.

**Keywords:** antibody array, atopic dermatitis, biomarker, blood, skin, stratum corneum, tape-stripping

## Abstract

The pathogenesis of atopic dermatitis (AD) involves various mediators, including cytokines and chemokines, which are produced by immune cells, such as dendritic cells and lymphocytes, and non-immune cells, such as epidermal cells. Several mediators, including thymus and activation-regulated chemokine (TARC), are used as biomarkers for AD severity and activity. However, additional local and systemic biomarkers of AD are required.

**Methods:**

This study will include 10 male patients with AD and 5 healthy adult males (age range: 20–80 years). The Eczema Area and Severity Index will be used to objectively evaluate the clinical findings. In addition, the severity of eruptions will be assessed on a 5-point scale by scoring symptoms (erythema, edema/papules, oozing/crusting, excoriation, lichenification, and xerosis), and the total intensity will be calculated by adding the symptom scores together. Subjective symptoms will be assessed using a peak pruritus numerical rating scale. Laboratory tests, including measurements of peripheral eosinophil count and serum total immunoglobulin E, TARC, and lactate dehydrogenase levels, will be performed. Using blood samples and extracts of stratum corneum samples obtained by tape stripping, we will conduct an exploratory analysis of protein expression using an antibody array to identify mediators whose levels are significantly altered in patients with AD. After 4 to 8 weeks, blood samples and stratum corneum samples will be collected again from AD patients. Moreover, we will examine whether the candidate proteins can be quantified using enzyme-linked immunosorbent assays.

**Discussion:**

This is an important study exploring potential local and systemic biomarkers of AD. The results of this study will be clinically meaningful for the discovery of new biomarkers for diagnosing and assessing the severity of AD.

## 1. Introduction

Atopic dermatitis (AD) is a common, chronic inflammatory skin disease. It mainly causes itchy eczema, which repeatedly goes into remission and then worsens.^[1–3]^ The pathomechanisms of AD are associated with skin barrier impairment and atopic background.^[1,2]^ The horny layer, the outermost layer of the epidermis that consists of keratin and filaggrin, is very tough. Loss-of-function mutations in filaggrin, which is crucial for the maintenance of skin barrier function, have been observed in patients with AD.^[4,5]^ Due to the impairment of the skin barrier function, several external stimuli are likely to affect the skin of patients with AD. Upon exposure to external stimuli, several epidermal-derived cytokines such as thymic stromal lymphopoietin, interleukin (IL)-33, and IL-25 are released and induce type-2 immune responses, leading to atopic skin inflammation.^[6–8]^ In the acute phase of AD, many type 2 T-helper cells (Th2 cells), which produce cytokines, including IL-4, IL-5, and IL-13, migrate to lesion sites, suggesting that Th2 cells play an important role in the early inflammation observed in AD. However, the number of Th1 cells, which produce interferon-γ, increases during the chronic phase.^[1,2]^ Recent studies have reported that IL-22-producing Th22 cells and IL-17-producing Th17 cells are also involved in the pathogenesis of AD.^[2,9,10]^

Several mediators have been used as biomarkers for AD severity and activity.^[11,12]^ Potential local and systemic biomarkers of AD include cytokines and chemokines, such as the levels of the Th2-related cytokine IL-13,^[13,14]^ the Th2-related chemokine C-C motif ligand (CCL)17/thymus and activation-regulated chemokine (TARC),^[15,16]^ CCL22/macrophage-derived chemokine,^[17,18]^ and the Th22-related cytokine IL-22^[17,19]^ in the blood and skin. However, further local and systemic biomarkers of AD are required. In particular, we need to identify a biomarker that is less invasive and can be used to evaluate the severity of AD.

In this study, we are going to use blood and stratum corneum samples collected from AD patients and healthy adults for antibody array screening. We will search for novel biomarkers and mediators associated with the severity and activity of AD and then examine whether the levels of the candidate proteins can be quantified.

## 2. Methods

### 2.1. The study design

This is a single-center, prospective, observational study, in which all samples and information will be obtained in Japan. Antibody array screening will be performed using blood and stratum corneum samples collected from patients with AD, that is, patients who meet the diagnostic criteria of the Japanese Dermatological Association, and healthy adults.

### 2.2. Eligibility criteria

Between the date of approval and June 30, 2023, 10 male patients with AD aged 20 to 80 years who had been diagnosed by a dermatologist as meeting the diagnostic criteria of the Japanese Dermatological Association^[1]^ and 5 adult males aged 20 to 80 years who have not previously had AD, asthma, or chronic skin diseases and have no family history of AD will be enrolled. PAD patients with AD and skin infections and those who have taken immunosuppressive drugs within 4 weeks of sample collection will be excluded.

### 2.3. Evaluation of clinical findings

As shown in Table 1, the eczema area and severity index will be used to objectively evaluate clinical findings. The severity of eruptions at lesional sites will be assessed on a 5-point scale (0, none; 1, very mild; 2, mild; 3, moderate; and 4, severe) by scoring the following symptoms: erythema, edema/papules, oozing/crusting, excoriation, lichenification, and xerosis. The total intensity will be calculated by adding the scores for each symptom, as reported previously.^[6,7,20]^ Subjective symptoms will be assessed using the Peak Pruritus Numerical Rating Scale. The Peak Pruritus Numerical Rating Scale is a single self-rated item, which was designed as a tool for measuring peak pruritus, or the worst itching, experienced over the previous 24 hours based on the following question: “On a scale of 0 to 10, with 0 being ‘no itch’ and 10 being ‘worst itch imaginable,’ how would you rate your itch at its worst during the previous 24 hours?”.^[21]^

**Table 1:** Outcome measures and time points of assessment.

		Visit 1	Visit 2
Week		0	4–8
Day		Day 0	Day 28–56
Eligibility	X		
Informed consent	X	(X)	
Patient background information collection		X	
**Objective clinical evaluations**			
EASI		X	X
Severity of the lesion		X	X
**Subjective clinical evaluation**			
PP-NRS		X	X
Blood tests			
Serum total IgE		X	X
Serum TARC		X	X
Serum LDH		X	X
Peripheral eosinophil count		X	X
Stratum corneum collection		X	X
Measurement of biomarkers (blood and stratum corneum)		X	X

EASI = Eczema Area and Severity Index, IgE = immunoglobulin E, LDH = lactate dehydrogenase, PP-NRS = Peak Pruritus Numerical Rating Scale, TARC = thymus and activation-regulated chemokine.

### 2.4. Collection of samples

We will collect stratum corneum samples from patients with AD, including both affected and unaffected sites. No topical substances will be applied to the collection sites 12 hours prior to collection. We will also collect stratum corneum samples from the skin of healthy individuals. The stratum corneum samples will be obtained using the tape-stripping method, in which the stratum corneum is sampled non-invasively by applying and removing adhesive tape (24 mm × 130 mm, Sellotape^®^; Nichiban Co., Ltd, Tokyo, Japan), as reported previously.^[6,7,20]^ After the tape is applied, a degree of pressure is applied and the tape is removed. In this study, the first and second pieces of tape will be discarded, and tape samples 3 to 22 will be collected, stuck to the front and back of glass slides (Matsunami Glass, Osaka, Japan), and stored at -40 °C. We will shred the stratum corneum tape samples and soak them in 0.2% Tween 20/phosphate-buffered saline. The mixture will be then transferred to a centrifuge tube, sonicated on ice, and centrifuged repeatedly to concentrate the protein fraction.

We will measure peripheral eosinophil count and serum total immunoglobulin E (IgE), thymus and activation-regulated chemokine, and lactate dehydrogenase levels. In addition, we will collect 10 ml of blood from each AD patient and separate the serum and plasma. After 4 to 8 weeks, blood and stratum corneum samples will be collected again from patients with AD. Blood and stratum corneum samples will be collected from the healthy adults. Protein extracts from the stratum corneum and blood samples will be subjected to antibody array screening (RayBio® Label-Based Human Antibody Array L-1000, RayBiotech, Inc.) to comprehensively examine protein expression, and these examinations will be outsourced to Cosmo Bio Co., Ltd. We will search for mediators whose levels are significantly altered in the AD patient group compared with those in the healthy group. Some proteins whose levels are significantly altered in the AD patient group will be subjected to enzyme-linked immunosorbent assays.

### 2.5. Statistical analysis

This is an exploratory observational study, and the sample size was based on the capacity of the institution during the study period. The primary endpoint will be analyzed by comparing the values of mediators between the AD patient group and the healthy control group at baseline. Blood samples and stratum corneum samples will be compared using Wilcoxon test. However, a parametric test method can be used depending on the distribution. Mediators that are significantly altered between groups will be compared between visit 1 (baseline) and visit 2 according to AD severity. We will analyze the data based on per-protocol analysis. The significance level will be set at 5% (2 sided). In the case of multiple tests, the Bonferroni correction will be performed. Descriptive analyses of the participants at the time of enrollment will be conducted.

### 2.6. Ethical consideration

This study complies with the Declaration of Helsinki, and the Ethical Guidelines for Medical and Health Research Involving Human Subjects has been approved by the institutional ethics committee (ERB-C-2131) and has been registered in the Japan Registry of Clinical Trials (jrct1050210175). All study participants will provide written informed consent before participating in the study.

## 3. Discussion

The aim of this study is to explore the potential local and systemic biomarkers of AD. Recently, the treatment of AD has markedly advanced; for example, biologics and Janus kinase inhibitors have been developed. If this study is successful, it may be possible to provide a more appropriate treatment by evaluating the severity and activity of AD locally and systemically. Moreover, as the stratum corneum samples will be collected using the tape-stripping method, we may be able to develop a method that allows the quantification of disease severity in a noninvasive manner.

This study will only include males between the ages of 20 and 80 years, because the hormonal changes caused by menstruation may make the results of the protein expression analysis difficult to interpret if females were included. One limitation of this study is that it will only include Japanese participants. Recently, it has been reported that Asian patients with AD exhibit markedly elevated IL-17 levels. Therefore, the results of this study may differ from those in non-Asian patients. With help from researchers worldwide, we would like to investigate whether potential local and systemic biomarkers are suitable for AD patients of different races in the future.

## Author contributions

All authors have read and agreed to the published version of the manuscript.

**Conceptualization:** Ayano Maruyama, Risa Tamagawa-Mineoka.

**Investigation:** Hiromi Nishigaki, Koji Masuda.

**Writing – original draft:** Ayano Maruyama, Risa Tamagawa-Mineoka, Norito Katoh.

**Writing – review & editing:** Ayano Maruyama, Risa Tamagawa-Mineoka, Hiromi Nishigaki, Koji Masuda, Norito Katoh.
